# Advancing liquid scintillation spectra deconvolution for radiological characterization: comparative techniques for multi-isotope activity assessment

**DOI:** 10.1007/s00216-026-06392-7

**Published:** 2026-02-20

**Authors:** M. Pérez-Baeza, S. Carlos, D. Ginestar, M. Sáez-Muñoz, S. Martorell

**Affiliations:** 1 Laboratorio de Radiactividad Ambiental, Universitat Politècnica de València, Cami de Vera s/n, València, 46022 València Spain; 2 Departament d’Enginyeria química i Nuclear, Universitat Politècnica de València, Cami de Vera s/n, València, 46022 València Spain; 3 Institut de Matemàtica Multidisciplinar, Universitat Politècnica de València, Cami de Vera s/n, València, 46022 València Spain

**Keywords:** Liquid scintillation, Spectra deconvolution, Activity determination, Beta emitters

## Abstract

In order to effectively control the level of radioactivity, faster analysis processing times are required. Therefore, new protocols must be developed and existing ones updated. Mathematical deconvolution methods, such as those based on adjusting the shape of the individual spectra of the isotopes, can be applied to separate these spectra, allowing a faster identification and quantification of the radioisotopes of interest. In this paper, several techniques based on Fourier series, Laguerre polynomials, Legendre polynomials, and Savitzky–Golay filter are used for spectral filtering, while multivariate statistical calibration techniques, such as least squares and partial least squares regressions, are used for spectral deconvolution and the prediction of the activity of beta emitters measured by liquid scintillation counting ($\boldsymbol{^{63}}$Ni, $\boldsymbol{^{55}}$Fe, $\boldsymbol{^{14}}$C, $\boldsymbol{^{3}}$H, $\boldsymbol{^{89}}$Sr, and $\boldsymbol{^{90}}$Sr). The results obtained for all the techniques analyzed are accurate, considering the maximum relative biases obtained in comparison with the reference activities and the maximum reconstruction errors of the spectra. Different relative biases are observed in the calculation of the activities of each radioisotope under study. Generally, the relative biases obtained are less than 20% for all techniques applied in most of the test samples. However, this difference is not so evident in terms of the reconstruction of the spectra, obtaining a maximum error of about 4.5%. Among all the techniques applied, the combination of Legendre polynomials or Laguerre polynomials and partial least squares regression offers the best results.

**Supplementary Information:**

The online version contains supplementary material available at https://doi.org/10.1007/s00216-026-06392-7.

## Introduction

The radiological characterization of different matrices (air, water, soil, and biota) is an important task that must be carried out during nuclear power plant operation to prevent the population and environment from exposure to ionizing radiation. Furthermore, the decommissioning of nuclear power plants will also require the rapid radiological characterization of new radioisotopes, not contemplated in the existing Environmental Radiological Surveillance Programmes, for a large quantity of complex waste and environmental samples. Radioactivity laboratories must be able to quickly identify and quantify mixtures of alpha and beta emitters present in contaminated samples. Therefore, new protocols are needed to control the level of radioactivity more effectively and quickly. These protocols can also be applied to new areas, such as radiological characterization for the segregation and valorization of NORM waste or in the case of radiological emergencies.

When rapid information on radioactivity levels is needed, such as in the case of a radiological emergency, the determination of gross alpha and gross beta indices is a useful screening method for detecting contamination from alpha and beta emitters [1, 2]. For example, some researchers have proposed direct measurement of water samples for these situations [3, 4]. However, the total alpha and beta indices do not allow to distinguish the isotopes involved. The quantification of specific alpha and beta emitters requires their isolation before the measurement by liquid scintillation spectrometry or other techniques.

In order to select the most optimal separation method, it is necessary to know which isotopes are present in the sample. Methods for analyzing water samples usually take a few hours, but more complex matrices can take days or even weeks to analyze. For example, a rapid method for emergency situations to determine radiostrontium in milk can take between 5 and 15 h [5], while a traditional method can take about 25 days, until achieving the secular equilibrium between $^{90}$Sr and $^{90}$Y [6].

The radiochemical separation of alpha and beta emitters may sometimes not be appropriate in a radiological emergency due to the time required for sample analysis. For this reason, the authors propose a straightforward approach to identify and quantify alpha and beta emitters measured by liquid scintillation spectrometry employing deconvolution techniques, with the ultimate goal of developing new, fast, and reliable radiological characterization methods, which can also be used for radiological emergencies. This preliminary study has been performed for the most relevant beta emitters ($^{63}$Ni, $^{55}$Fe, $^{14}$C, $^{3}$H, $^{89}$Sr, and $^{90}$Sr) from the decommissioning of nuclear power plants.

Liquid scintillation spectrometry is widely used in radioactivity laboratories to measure beta emitters and, to a lesser extent, alpha emitters. This technique is highly efficient due to its 4π geometry, allows the analysis of liquid and solid samples as long as the samples can be dissolved or suspended in the scintillating liquid, and measures both the number of decay events (quantification) and the energy of the radiation (spectroscopy), since the intensity of the emitted light is proportional to the energy of a radioactive event. This allows a spectrum to be visualized, showing the peaks of radioisotopes in the sample. However, the lower energy resolution, compared to other techniques such as alpha spectrometry with silicon detectors, together with the continuous nature of beta emissions, sometimes prevents the correct quantification of the radioisotopes of interest because of the overlap between them. This overlapping can sometimes be avoided by performing a prior radiochemical separation, which usually involves a long analysis time and a significant economic expense in terms of material and reagents. For example, in the case of $^{90}$Sr determination in food and other environmental samples, most authors propose the separation of radiostrontium from matrix interferences [6–8]. However, spectra overlapping can occur due to a not satisfactory or impossible radiochemical separation of radionuclides (e.g., when radioisotopes of the same chemical element are involved in the radiochemical separation, such as $^{89}$Sr and $^{90}$Sr). Therefore, the application of spectra deconvolution techniques is a useful, faster, and cost-effective method to identify and quantify the activity of the radioisotopes of interest as the authors of this paper previously proposed for $^{89}$Sr and $^{90}$Sr spectra isolation [9].

**Table 1:** Radionuclides main characteristics: main disintegration mode, emission energy (*Q*, keV), half-life ($T_{1/2}$, years), and daughters. Data obtained from Laboratoire National Henri Becquerel

Isotop	Disintegration	*Q* (keV)	$T_{1/2}$ (years)	Daughters
$^{55}Fe$	Electron capture	231.12	2.747	$^{55}Mn$ (stable)
$^{63}$Ni	$\beta ^-$	66.98	98.7	$^{63}$Cu (stable)
$^{14}$C	$\beta ^-$	156.476	5700	$^{14}$N (stable)
$^{3}$H	$\beta ^-$	18.591	12.312	$^{3}$He (stable)
$^{89}$Sr	$\beta ^-$	1495.1	0.14	$^{89}$Y (stable)
$^{90}$Sr	$\beta ^-$	545.9	28.8	$^{90}$Y, $^{90}$Zr (stable)

Over the last 20–30 years, different techniques have been applied to separate the spectra of different radioisotopes such as window techniques [10] that are based on establishing different counting windows to determine the individual activity of each radioisotope, or multivariate statistical calibration techniques that can be used to predict the activity of unknown samples from the spectra of the isotopes of interest with known activity, using partial least squares regression (PLS) or principal component analysis (PCA) systems [11–13]. Another option is the use of discrete functions such as the Fourier series to fit the shape of the individual spectra of the isotopes to separate each signal [14–16]. Legendre polynomials and Laguerre polynomials [17] or Savitzky–Golay filter [18, 19] are also used for these purposes. The authors [9] previously worked on the deconvolution of spectra based on fitting the spectra $^{89}$Sr and $^{90}$Sr mixtures using discrete functions such as Fourier series, Legendre polynomials, and Laguerre polynomials and determining their activity by means of a least squares procedure. Neuer [20] used a deconvolution technique based on maximum likelihood, using a surrogate model and performing simulations with the GEANT4 code to obtain the spectrum of $^{90}$Sr when masked by $^{40}$K, $^{131}$I, or $^{226}$Ra, respectively. Bi [21] proposed a reconstruction and analytical method for seawater radioactive gamma spectrum based on the Gaussian response matrix and the boosted weighted non-negative least squares (Boosted-WNNLS). Borovcová [22] has also used Gaussian functions in mass spectrometry applications, which could be an effective technique to determine radioactive isotope spectra. Non-target screening methods by high-resolution mass spectrometry are powerful approaches in analytical chemistry for identifying unknown compounds in complex samples. These methods also require data processing algorithms. Renner and Reuschenbach [23] reviewed the strengths and weaknesses of different algorithms for non-target screening. In this reference, the authors address the issues of uncertainty and data quality, emphasizing the importance of incorporating confidence intervals and assessing the quality of raw data. Furthermore, they propose the use of standardized statistics and open-access data exchange platforms.

Currently, there are also techniques based on artificial intelligence that allow the spectra deconvolution. For example, Pilato [24] used a neural network in gamma spectrometry to determine the activity of different radioisotopes. In [25], a neural network was used for rapid determination of $^{89}$Sr/$^{90}$Sr activity in water samples and aerosol filters from a nuclear power plant. More recently, Li [26] presented a radionuclide identification method using a one-dimensional convolutional neural network, providing good results for mixtures of low activity for industrial and medical applications. Other authors also employed simple and more evolved neural networks, known as *deep learning*, to detect and quantify peaks in nuclear magnetic resonance spectra, to identify bacterial species using single-particle mass spectrometry, and for other industrial applications [27–31].

In this work, a study on the comparison of different spectral deconvolution methods to correctly quantify the activity of beta emitters from nuclear power plant decommissioning measured by liquid scintillation counting has been conducted. Several techniques have been used to undertake spectral deconvolution of mixtures. In this work, the authors propose the use of Legendre and Laguerre polynomials as filtering techniques alternative to Savitzky–Golay and Fourier methods already used in spectral deconvolution of isotope mixtures [14, 19]. In addition, multivariate statistical calibration techniques, such as least squares and partial least squares regressions, were used for spectral deconvolution and activity prediction of the radioisotopes of interest. A comparison of the performance of all these techniques was analyzed. This will enable the community to advance in the field of spectral deconvolution methods and improve the reliability of results.

## Experimental activity determination

In order to classify and store nuclear waste, a radioactivity inventory of materials from decommissioned nuclear facilities is necessary. In this sense, apart from the reactor cores, the main source of radioactivity in the reactor is the graphite and the construction materials. The irradiated reactor graphite, the concrete shield, or the metal materials such as aluminum alloy, steel, and lead are radioactive due to the presence of various nuclides, including $^{3}$H, $^{14}$C, $^{36}$Cl, $^{41}$Ca, $^{55}$Fe, $^{60}$Co, $^{63}$Ni, $^{90}$Sr, $^{133}$Ba, $^{137}$Cs, $^{152}$Eu, $^{154}$Eu, and some transuranic elements. Gamma emitters (e.g., $^{60}$Co, $^{133}$Ba, $^{137}$Cs, $^{152}$Eu, and $^{154}$Eu) can easily be determined by gamma spectrometry, while alpha and beta emitters (e.g., $^{3}$H, $^{14}$C, $^{36}$Cl, $^{41}$Ca, $^{55}$Fe, $^{63}$Ni, $^{90}$Sr, and the transuranic elements) require the isolation of the radionuclides from the matrix and from the other radionuclides for their determination [32]. In this study, three sets of mixtures containing the most relevant beta emitters ($^{63}$Ni, $^{55}$Fe, $^{14}$C, $^{3}$H, $^{89}$Sr, and $^{90}$Sr) from the decommissioning of nuclear power plants have been selected. These mixtures ($^{55}$Fe/$^{63}$Ni, $^{14}$C/$^{3}$H, and $^{89}$Sr/$^{90}$Sr) have been used to analyze the performance of the different deconvolution techniques proposed in “Isotopes activity estimation from a mixture spectrum.” Some of the main characteristics of these radionuclides are shown in Table 1.

### Reagents and solutions

The $^{55}$Fe (75.5±2.3 kBq/g), $^{63}$Ni (41.3±0.9 kBq/g), $^{89}$Sr (196.6±3.0 kBq/g), $^{90}$Sr/$^{90}$Y (39.6±0.6 kBq/g), $^{14}$C (4.68±0.16 kBq/g), and $^{3}$H (29.0±0.7 kBq/g) standard solutions were from Physikalisch-Technische Bundesanstalt (Germany). The appropriate amount of the standard solution was dissolved in 0.1 M HCl to prepare intermediate dilutions for the calibration standard solutions and the test isotope mixtures. The decay of the isotopes under study was taken into account when calculating the activities of all samples.

All reagents were of analytical grade. Ammonium hydroxide 25%, hydrochloric acid 37–38%, and nitric acid 65% were from J. T. Baker - Avantor Inc. (Poland). Strontium nitrate was from EMSURE®-Merck Millipore (Germany). Iron(III) chloride hexahydrate and sodium carbonate were from PanReac AppliChem- ITW Reagents (Spain). Integrity+Trace water purification equipment (Androna, Latvia) was used to prepare the solutions.

The 0.1 M HCl solution was prepared by dissolving the appropriate amount of hydrochloric acid 37–38% in ultrapure water. The 1.5 M Na$_{2}$CO$_{3}$ solution was prepared by dissolving the appropriate amount of sodium carbonate in ultrapure water. Strontium carrier (20 mg/mL) solution was prepared by dissolving the appropriate amount of strontium nitrate in HNO$_{3}$ 0.065% medium, and iron carrier (5 mg/mL) solution was prepared by dissolving the appropriate amount of iron(III) chloride hexahydrate in ultrapure water.

For the preparation of the calibration standard solutions and the test isotope mixtures, 20-mL polyethylene vials (PerkinElmer Inc.) were used. The water was HPLC grade (J. T. Baker), and Ultima Gold$^{TM}$ LLT (PerkinElmer Inc.) was used as scintillation cocktail. The sample-to-scintillator ratio was 8:12, as the authors previously proposed for gross alpha and gross beta determination by direct measurement in liquid scintillation counting [4]. Different proportions can be used depending on the laboratory’s needs. However, it should be noted that the deconvolution method must be calibrated under the same conditions as the samples and blanks that are going to be analyzed.

**Fig. 1:**
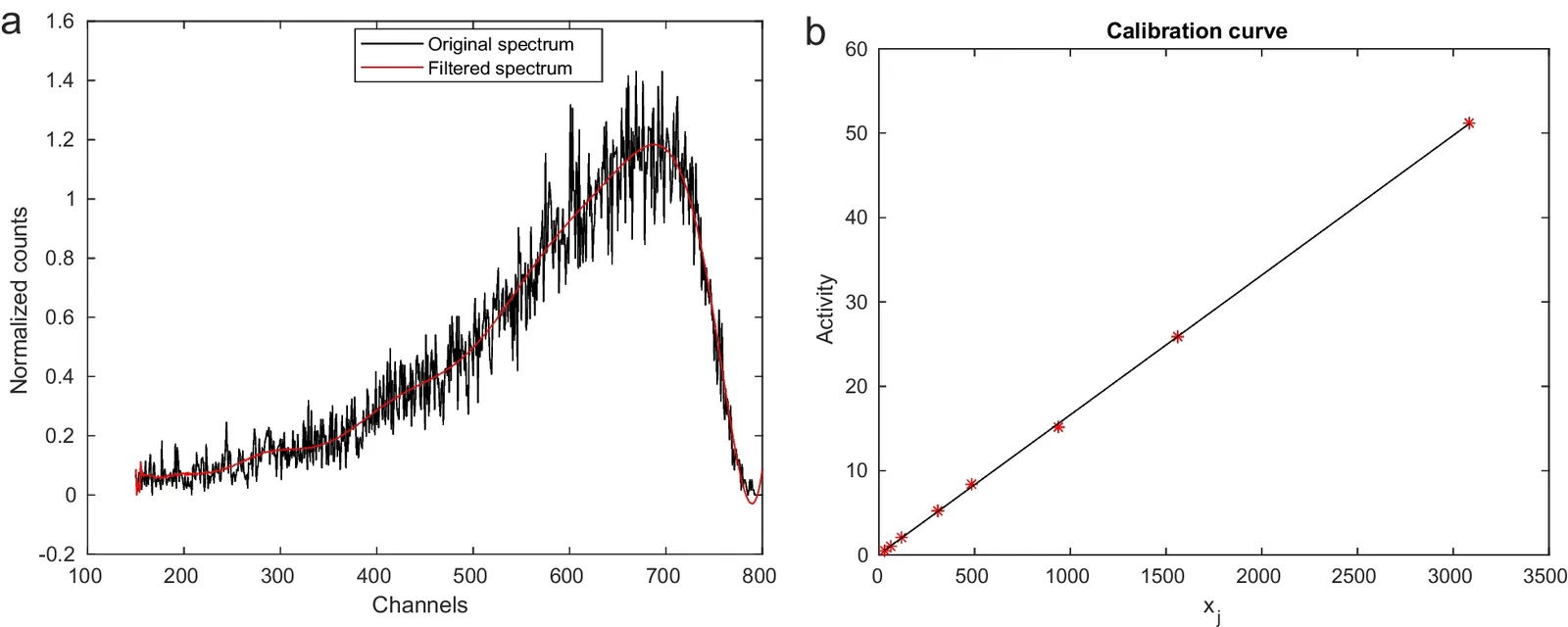
Spectrum filtering and calibration curve

Eight concentration levels for calibration standard solutions were prepared in the activity range from 0.5 to 50 Bq by dilution of the intermediate standards in water. To achieve this, the required volume of water was added to a vial to bring the total volume to 8 mL with the corresponding standard. Then, 12 mL of scintillation cocktail (Ultima Gold$^{TM}$ LLT) was added. The vials were shaken for 1 min and placed in the equipment until measured. Due to the equilibrium between $^{90}$Sr and its decay product, $^{90}$Y, $^{90}$Sr must be isolated from $^{90}$Y. Then, a fast radiochemical separation of both elements is necessary in the case of the standard of $^{90}$Sr/$^{90}$Y. In brief, the first step is to adjust the pH to 8–9 using NH$_{4}$OH to precipitate iron and actinides. Then, $^{90}$Sr is isolated from $^{90}$Y by filtration. Next, strontium is precipitated using 1.5 M Na$_{2}$CO$_{3}$. After centrifuging, the precipitate is dissolved in 0.1 M HCl. Finally, a vial is prepared in the same way as the other calibration standards. This involves adding 8 mL of the solution obtained from the radiochemical separation process and 12 mL of scintillation cocktail to a vial. Chemical recovery of the radiochemical separation is obtained by atomic absorption spectrometry, using the remaining solution from the radiochemical separation process. A blank was also prepared in the same way, that is, 8 mL of HPLC water and 12 mL of scintillation cocktail were added to a vial. The tables in the supplementary material show the corresponding data for calibration standard solutions.

For $^{14}C/^{3}H$ and $^{63}Ni/^{55}Fe$ mixtures, up to 15 concentration levels for test isotope mixtures were prepared in the activity range from 0.5 to 50 Bq and different proportions of each isotope (from 1:1 to 1:10) by dilution of the intermediate standards in water, using the same procedure as that used to prepare calibration standard solutions. For $^{89}Sr/^{90}Sr$, nine mixtures were prepared following the same procedure used for the preparation of the $^{90}$Sr calibration standards. The corresponding data for the test isotope mixtures can be found in the supplementary material tables.

### Instrumentation

The 1220 QUANTULUS$^{TM}$ Ultra-Low Level Liquid Scintillation Spectrometer (PerkinElmer Inc.) was used to measure the calibration standards, the mixtures of the isotopes, and the blank.

A liquid scintillation spectrometer is a detector that can measure extremely low concentrations of artificial, cosmogenic, and natural radionuclides. It has two types of shielding, active and passive, which significantly reduce the environmental background. It works with two coincident photomultiplier tubes that can collect the radiation emitted by the sample and convert it by a suitable electronic circuit into an electrical signal that provides information related to the isotopes’ activity, which is proportional to the incident radiation under the defined working conditions. The quantification of the activity requires measuring the sample counts, knowing the blank counts, and determining the counter efficiency, which is obtained by calibrating the equipment with a known activity standard for each isotope being studied.

The WinQ equipment acquisition software (PerkinElmer Inc.) was used to establish the parameters and necessary measurement conditions for each radionuclide, and the EasyView analysis software (PerkinElmer Inc.) was used to obtain the data. The measurement protocol used in the measurements was the one pre-established in the equipment for low-energy beta emissions. Samples were generally measured for 180 min (three cycles of 60 min each) except for $^{89}$Sr/$^{90}$Sr mixtures that were measured for 120 min (three cycles of 40 min each) due to $^{90}$Y growth, daughter of $^{90}$Sr. The evaluation of the sample quenching was performed using the spectral quenching parameter of the external standard (SQP(E)). The SQP(E) values for all calibration standards and mixtures ranged from 701 to 714, with a variation of less than 2%, which is negligible. Therefore, the influence of this parameter has not been evaluated in this study. However, future studies are planned to examine the impact of this parameter on activity predictions for samples with different SQP(E) values. The selected counting window for all spectra was set to 1–1024.

## Isotopes activity estimation from a mixture spectrum

The spectra deconvolution of a given mixture is performed in two steps. First, a calibration process is needed to obtain a normalized spectrum for each one of the isotopes present in the mixture. The second step consists of the deconvolution of the spectrum corresponding to the mixture using the normalized spectra obtained for the different isotopes. We will use a deconvolution technique based on a least squares fitting and a partial least squares regression.

### Normalized average spectrum

For a dilution with a mixture of known isotopes, it is necessary to prepare a battery of dilutions with only one of the isotopes with known activity, obtaining *K* spectra with activities $a_1, \ldots , a_K$. The spectrum corresponding to the background is subtracted from each of the spectra, and a filtering process is used. In Fig. 1a, a typical spectrum is shown together with the result of the filtering process.

The filtered spectra $S_{f,j}=\left( s_1,\ldots ,s_c\right)$, where *c* is the number of channels of the spectrum, are normalized

$$\begin{aligned} S_{N,j}=\frac{S_{f,j}}{\sum _{i=1}^c s_i} \end{aligned}$$

and a normalized average spectrum

$$\begin{aligned} S_{av}=\frac{\sum _{j=1}^K S_{N,j}}{K} \end{aligned}$$

is obtained for each one of the isotopes of the mixture.

Finally, for each filtered spectrum of the battery, using a minimum least squares technique, we compute a coefficient $x_j$ such that

1 $$\begin{aligned} S_{f,j} = x_j S_{av}, \ \ \ j=1, \ldots K. \end{aligned}$$

Using the obtained coefficients $x_j$ for the different activities, $a_j$, of the isotope, a calibration curve

2 $$\begin{aligned} a_j = \alpha x_j + \beta \ , \end{aligned}$$

is obtained. A typical calibration curve for one isotope is shown in Fig. 1b.

### Spectrum filtering

Raw data extracted directly from the experimental measurements present some noise that must be eliminated to proceed with the spectra treatment. Filtering methods based on expansions in terms of orthogonal functions, Fourier approximation, and the Savitzky–Golay filter have been used in this work.

#### Orthogonal functions

The spectrum filtering based on orthonormal functions approximates the digital signal corresponding to the spectrum *S*(*n*), n=1,…,N as the expansion.

3 $$\begin{aligned} S(n) = \sum _{j=1}^{N_f} C_{j} \pi _j(n)\ , \end{aligned}$$

where $\pi _j(n)$ are discrete functions that are orthonormal, that is,

$$\begin{aligned} \langle \pi _i, \pi _j \rangle = \sum _{l=1}^N \pi _i(n)\, \pi _j(n) = \delta _{i,j} , \end{aligned}$$

where $\delta _{i,j}$ is the Kronecker delta function [33]. To construct these orthonormal functions, a set of generating functions

4 $$\begin{aligned} \left\{ \varphi _1(n), \ldots , \varphi {Nf}(n) \right\} , \end{aligned}$$

is selected and the orthogonal functions $\pi _j(n)$ are obtained performing the *QR* decomposition of the matrix defined by the set of generating function [33], and identifying $\pi _j(n)$ as the columns of the *Q* matrix. The coefficients of expansion Eq. 3 are

5 $$\begin{aligned} C_j = \langle \pi _j, S \rangle = \sum _{n=1}^N \pi _j(n) S(n)\ \end{aligned}$$

Two sets of orthogonal functions are considered: the Laguerre functions and the Legendre functions.

#### Laguerre functions

For continuous functions, using the usual scalar product

$$\begin{aligned} \langle f, g \rangle = \int _0^{+ \infty } f(t) g(t) \, dt \quad \end{aligned}$$

the set of generating functions

6 $$\begin{aligned} \left\{ (p t)^j e^{-pt} \right\} \ \ \ j=0,1,\ldots \ ; \ \ \ p>0\quad , \end{aligned}$$

is a complete set of functions, and it is possible to obtain an orthonormal set of functions, $La_j(t)$, orthonormalizing the set Eq. 6, [34].

In a similar way, we define the discrete Laguerre functions as the orthonormal set of functions obtained by orthonormalizing the set of discrete functions

7 $$\begin{aligned} \{ (np)^j e^{-pn} \}\ \ ; \ \ \ j=0,1,\ldots , Nf \ ,\ \ \ p >0 \quad , \end{aligned}$$

to define the discrete Laguerre functions $La_j(n)$.

#### Legendre functions

In order to construct the Legendre functions, we started from the standard Legendre polynomials, which are orthogonal functions on the interval [-1,1], $P_j(t)$ [34]. They satisfy the condition

8 $$\begin{aligned} \int _{-1}^{1} P_i(x) P_j(x) \, dx = \frac{2}{2 i + 1} \delta _{i,j} \quad \end{aligned}$$

Using the change of variables $x = 2 e^{-pt} -1$; p>0, allows the passage from the interval [-1,1] for the variable *x*, to the interval [0,+∞] for the variable *t*. In terms of the new variable, the orthogonality relation Eq. 8 becomes

9 $$\begin{aligned} \int _{0}^{+\infty } p e^{-pt} P_i(2 e^{-pt} -1 ) P_j(2e^{-pt}-1) \, dt = \frac{1}{2 i + 1} \delta _{i,j} \quad \end{aligned}$$

Introducing

$$\begin{aligned} Le_i(t) = \sqrt{p (2 i + 1)} e^{-\frac{p}{2}t} P_i(2 e^{-pt}-1) \quad , \end{aligned}$$

we have the following relation:

10 $$\begin{aligned} \int _{0}^{+\infty } Le_i(t) Le_j(t) \, dt = \delta _{i,j} \quad \end{aligned}$$

In a similar way, one defines the discrete Legendre functions as the orthonormal set of functions resulting from the orthonormalization of the generating set

11 $$\begin{aligned} \left\{ e^{-(2j+1) \frac{p}{2}n} \right\} \ \ ; \ \ \ j=0,1, \ldots , Nf \ \ ; \ \ \ p>0\quad , \end{aligned}$$

obtaining the discrete Legendre functions $Le_j(n)$.

#### Fourier method

Given a spectrum *S*(*n*), n=1,…,N, the Fourier filtering of this spectrum is obtained approximating [14],

12 $$\begin{aligned} S(n) = a + b (n-1) + \sum _{j=1}^{Nf} C_j \sin \left( \frac{\pi j (n-1)}{N-1}\right) \ , \end{aligned}$$

where

$$\begin{aligned} a= &   S(1)\\ b= &   \frac{S(N)- S(1)}{N-1}\\ C_j= &   \frac{2}{N-1}\sum \limits ^{N}_{n=1} \sin \left( \frac{\pi j (n-1)}{N-1}\right) \, (S(n)-a-b(n-1))\ \end{aligned}$$

Essentially, the spectrum *S*(*n*) is approximated by a discrete Fourier series considering only *Nf* harmonics. The term a+b(n-1) has been added in order to avoid possible negative fitted values near n=1 [14].

#### Savitzky–Golay filter

The Savitzky–Golay filter [18, 35] is widely used in spectroscopy for data smoothing, since it preserves the shape and main features of the signal. This filter is based on approximating subsets of adjacent data points by windowing the signal with a low-degree polynomial using linear least squares approximation. The value of the polynomial of the central point of the subsets is taken as the filtered value of the signal. If the signal data are equally spaced, analytical expressions can be derived for the solution of the linear least squares problem, making the application of the filter very efficient. In this work, we have used the Python function “savgol_filter()” from the SciPy library for the implementation of this filter. This function uses the window length and the degree of the polynomial used for the approximation as parameters.

### Least squares activities determination

Let us assume that we start from the spectrum of a given dilution that contains two known isotopes. The described technique can be easily extended if more than two isotopes are contained in the dilution.

The spectrum of the test isotope mixture is filtered with the same technique as the one used in the calibration process. From the calibration, we have filtered and normalized the spectrum of each of the isotopes $S_1(n)$ and $S_2(n)$. It is assumed that

13 $$\begin{aligned} S(n)= x_1 S_1 (n) + x_2 S_2 (n) \ \end{aligned}$$

The coefficients $x_1$ and $x_2$ are determined minimizing the error

$$\begin{aligned} \varepsilon \left( x_1, x_2\right) = \sqrt{\frac{\sum _{n=1}^N \left( S(n) - x_1 S_1(n)-x_2 S_2(n)\right) ^2 }{\sum _{n=1}^N S^2(n)}}\ \end{aligned}$$

In the implementation in Python performed in this work, the coefficients are obtained with a quasi-Newton method [36], initializing the coefficients as $\left( x_1,x_2\right) =(1,1)$. Particularly, the function “minimize()” from the *Scipy* library has been used.

**Fig. 2:**
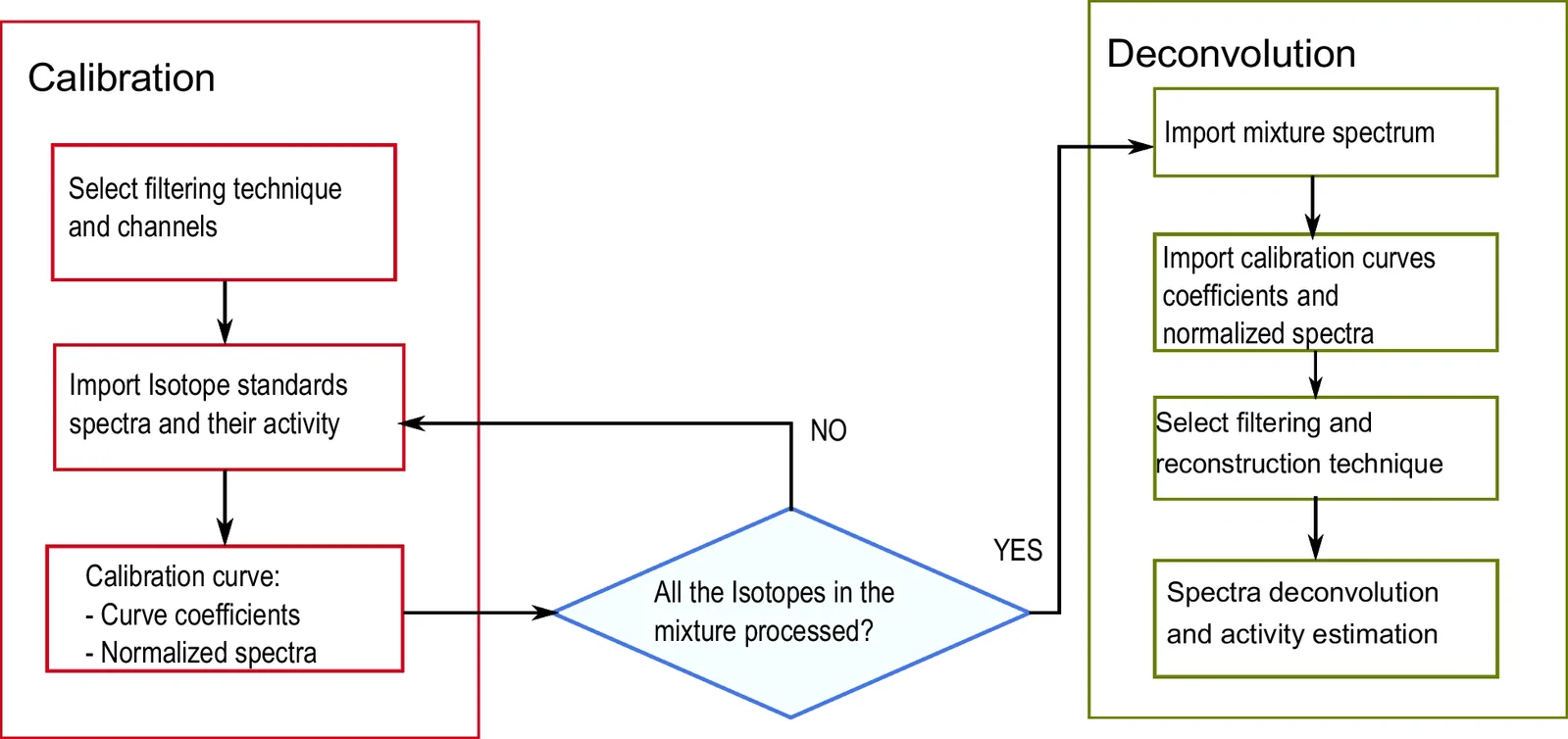
Methodology workflow

### PLS regression

Partial least squares regression is a multivariate data analysis widely used for spectra analysis in chemometrics [11, 37–39]. Considering *x* as the predictor and *y* as the response variable, respectively, this technique relies on a linear model of the form

14 $$\begin{aligned} y = b_0+b_1 x_1 + \cdots + b_m x_m + e\ , \end{aligned}$$

where the coefficients $b_0, \ldots , b_m$ have to be determined. These coefficients could be determined using the classical least squares technique, but this is not used in chemometrics because the predictor variables have a high correlation among them. Partial least squares (PLS) technique was introduced by Herman World [40, 41] in the field of economics, and his son [11] introduced this technique in chemometrics. Having $N_t$ observations of the predictor and the response variables, the linear model Eq. 14 is rewritten as

Y=XB+E

where $X\in \mathbb {R}^{N_t\times m}$, $Y\in \mathbb {R}^{N_t\times q}$, and *E* is an error matrix.

Variables *X* and *Y* are decomposed as

$$\begin{aligned} X= &   T P^T + E_x \,\\ Y= &   U Q^T + E_y \, \end{aligned}$$

where $E_x$ and $E_y$ are also error matrices. Matrices *P*, *Q*, *T*, and *U* have as many columns as the number of components considered, $a \le \min \left\{ m, q, n\right\}$. Matrices *T* and *U* are related by

U=TD+H

being *H* an error matrix and *D* the matrix with the regression coefficients of the model, which are calculated by maximizing the covariance between *T* and *U*.

In the implementation done for a mixture with two isotopes, a set of synthetic spectra is generated using the normalized spectrum of each one of the isotopes of the mixture as

15 $$\begin{aligned} S(n) = x_1 S_1(n) + x_2 S_2(n) \ \end{aligned}$$

For that, a minimum and a maximum activities are considered, $a_{\textrm{min}}$, $a_{\textrm{max}}$, and a two sets of $N_t$ random variables for the activities $a_1$ and $a_2$ are generated in the interval $\left[ a_{\textrm{min}}, a_{\textrm{max}}\right]$. The corresponding coefficients for the $x_1$ and $x_2$ for the synthetic mixtures are obtained using the calibration curves (Eq. 2). From this training set of mixture spectra, the regression coefficients of the model are obtained using the function “PLSRegression()” from the Python library “Scikit-learn.” This regression model is used to estimate the isotopes’ activities corresponding to a measured spectrum for a mixture. In our implementation, we have considered $N_t=500$ and a=2 components.

### Calculation methodology

Figure 2 summarizes the different steps that have to be undertaken to obtain the reconstructed spectra of the individual isotopes present in the mixture and the estimation of their activity. Calibration standards must be prepared and measured in the liquid scintillation spectrometer available in the laboratory. For the moment, the methodology is prepared for Quantulus 1220 spectra.

First of all, each isotope must be calibrated, selecting the channels in which the spectrum information appears and one of the filtering techniques available. Then, for each individual isotope, the individual standard spectra together with its associated activity have to be uploaded to the tool. Using these data, the calibration curve is performed, and the coefficients of such calibration and a normalized spectrum of the isotope are obtained and saved in two files. These steps are repeated as many times as isotopes we consider there exist in the mixture. Once all the isotopes are processed, the mixture reconstruction calculation is undertaken. To do this, a mixture spectrum measured with Quantulus 1220 is uploaded and filtered, using the same filtering technique as the one selected in the calibration phase. After that, the reconstruction method, LS or PLS, is selected and the tool calculates the spectra of each individual isotope present in the mixture, estimates their activity, and provides the error of the reconstruction.

## Numerical results

All of the techniques previously explained have been implemented in Python, as one of the objectives of the work is to develop a free tool that will be available to all of the environmental radioactive laboratories. Thus, after a brief sensitivity analysis of the parameters needed in each filter, the following values were selected as the most appropriate ones. For Laguerre and Legendre polynomials, the number of functions is $N_f=15$ and the parameter p=0.001. For the Fourier series, the number of harmonics considered is $N_f=15$. To obtain these values, a simple sensitivity analysis has been undertaken on $^{63}Ni$ and $^{55}Fe$ standards. Thus, $N_f$ has been changed in the range 10–30, and *p* between 0.1 and 0.001. The parameters for which the minimum error was obtained have been selected in this study. Finally, for the Savitzky–Golay filter, the window length is set to 20, and the order of the polynomial used to fit the samples is set to 2.

The individual radioactive standards ($^{55}Fe$, $^{63}Ni$, $^{3}H$, $^{14}C$, $^{89}Sr$, and $^{90}Sr$) were used to calibrate or test the methods, and then the spectra of the spiked mixtures prepared in the laboratory ($^{55}Fe$/$^{63}Ni$, $^{3}H$/$^{14}C$, and $^{89}Sr$/$^{90}Sr$) were used to validate them. The validation was performed with the analysis of the reconstruction error of each technique, and the assessment of the relative bias between experimental and estimated activities.

**Fig. 3:**
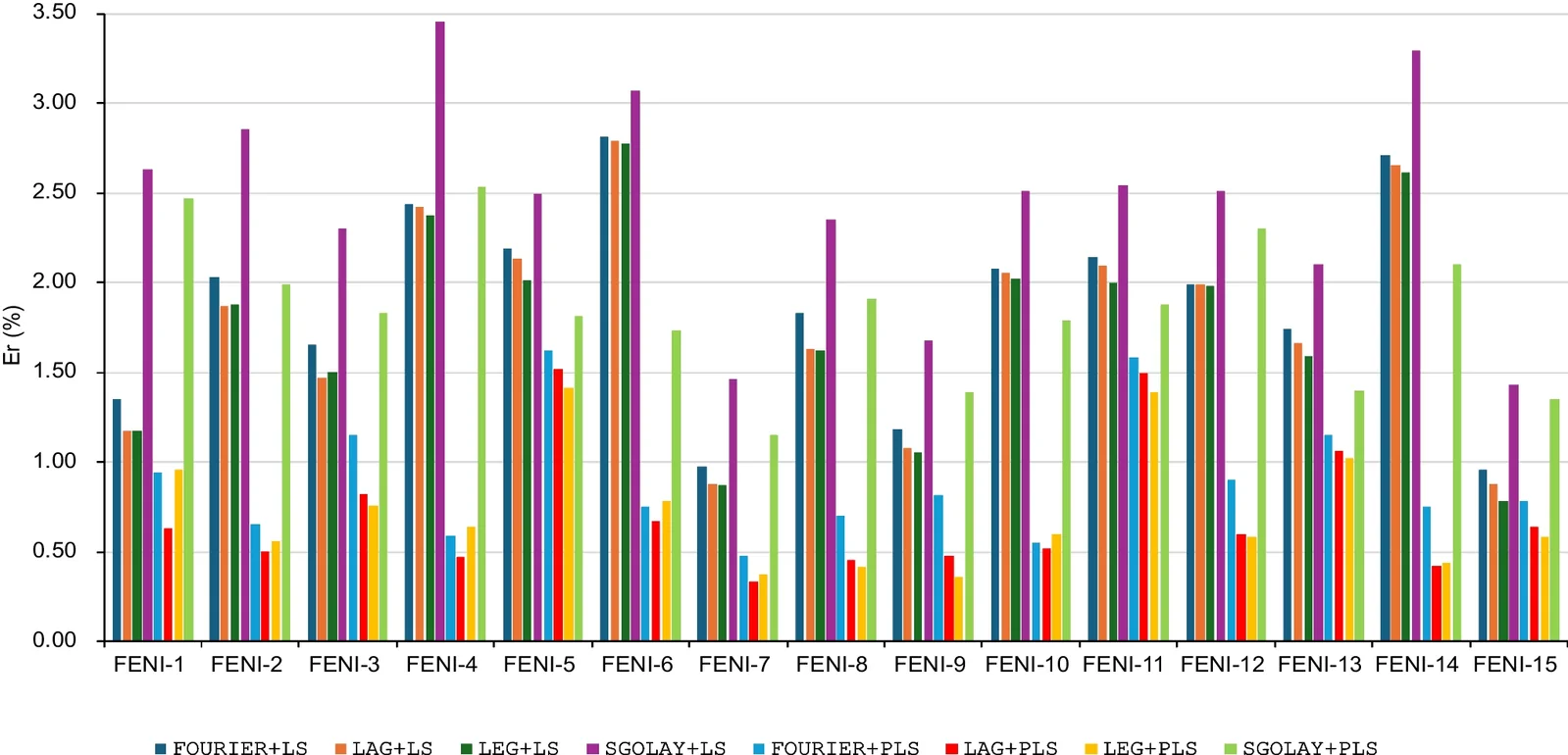
Spectra reconstruction errors (Er, %) obtained applying each deconvolution technique (least squares and partial least squares regressions) with the different filters (Fourier series, Laguerre polynomials, Legendre polynomials, and Savitzky–Golay filter) for all $^{63}Ni$/$^{55}Fe$ mixtures

**Fig. 4:**
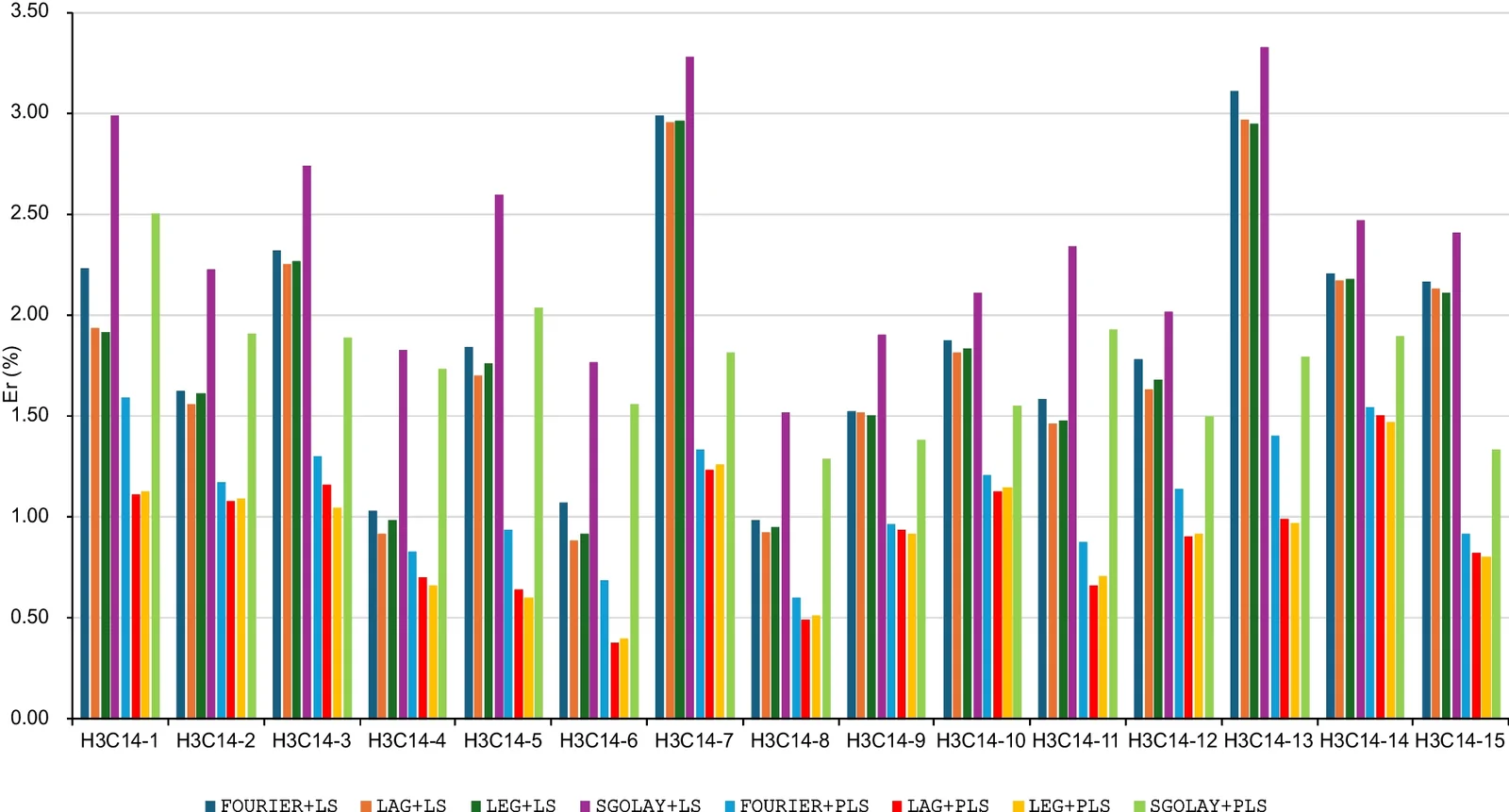
Spectra reconstructions errors (Er, %) obtained applying each deconvolution technique (least squares and partial least squares regressions) with the different filters (Fourier series, Laguerre polynomials, Legendre polynomials, and Savitzky–Golay filter) for all $^{14}C/^{3}H$ mixtures

**Fig. 5:**
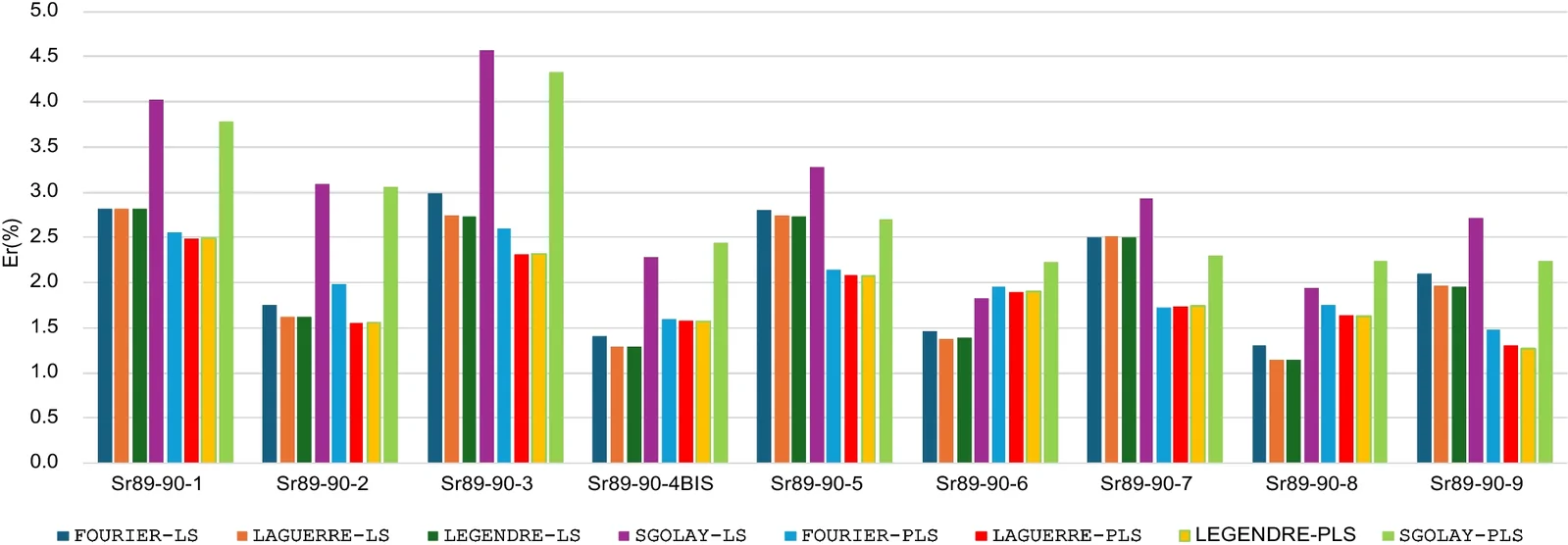
Spectra reconstructions errors (Er, %) obtained applying each deconvolution technique (least squares and partial least squares regressions) with the different filters (Fourier series, Laguerre polynomials, Legendre polynomials, and Savitzky–Golay filter) for all $^{89}Sr/^{90}Sr$ mixtures

Figures 3, 4, and 5 show the error in the spectra reconstruction obtained using each technique for all the isotope mixtures of $^{63}Ni$/$^{55}Fe$, $^{14}C/^{3}H$, and $^{89}Sr/^{90}Sr$, respectively. It can be noted that, in all cases, the error obtained is quite low. For all cases, spectral reconstruction errors less than ± 5% are obtained.

In particular, for $^{63}Ni$/$^{55}Fe$ mixtures (see Fig. 3), the maximum error obtained is 3.45% for the mixture FENI-4. This error was obtained using Savitzky–Golay for filtering and least squares (LS) regression to perform the spectra reconstruction. It can also be observed that using the partial least squares (PLS) regression, the errors obtained in the reconstruction are always lower than those calculated using LS for any of the filter techniques proposed in this work. The minimum error calculated in $^{63}Ni/^{55}Fe$ mixtures is 0.33% using the Laguerre polynomials and PLS for the mixture FENI-7. Taking into account all the samples analyzed, the minimum average error in the reconstruction for the test isotope mixtures is obtained using PLS and the Laguerre or Legendre polynomials with 0.71% and 0.72%, respectively.

**Fig. 6:**
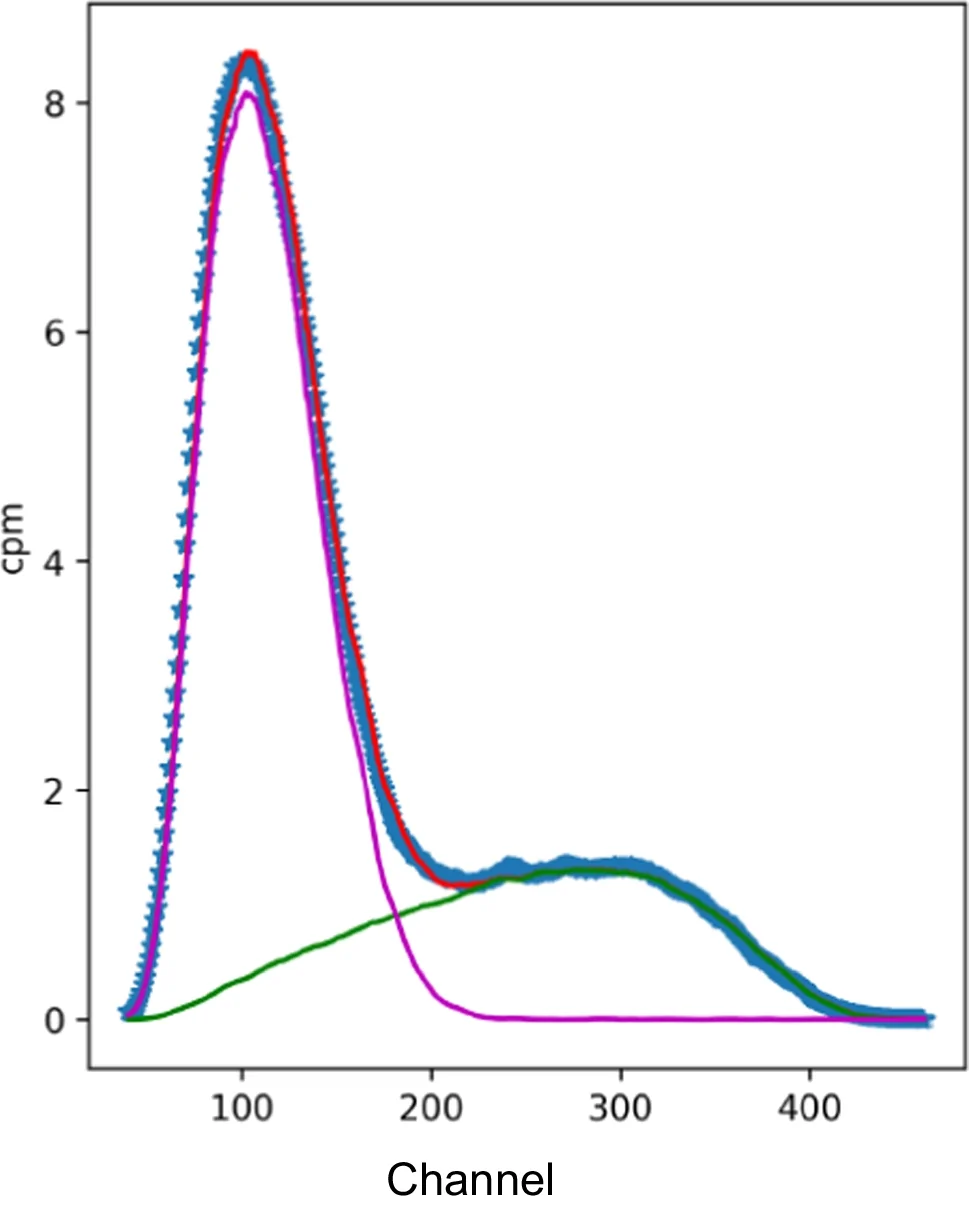
Experimental and reconstructed spectra for H3C14-7. The experimental (  ) and reconstructed (  ) spectra of the mixture, together with the individual spectra of $^{14}C$ (  ) and $^{3}H$ (  ) are shown

Regarding the $^{14}C/^{3}H$ mixtures, Fig. 4 shows the errors in the spectra reconstruction calculated for each technique. In this case, the maximum error found is 3.28% for the mixture H3C14-7, using the Savitzky–Golay filter and PLS. Figure 6 shows the experimental and reconstructed spectra for this mixture. The minimum error value was found in the mixture H3C14-6 using the Laguerre polynomials and PLS. As in the previous case, to assess the performance of each technique in all the mixtures considered, the average error in the reconstruction has been calculated, observing that the minimum value has been obtained using the Laguerre or Legendre polynomials and PLS with 0.92% and 0.91%, respectively.

**Fig. 7:**
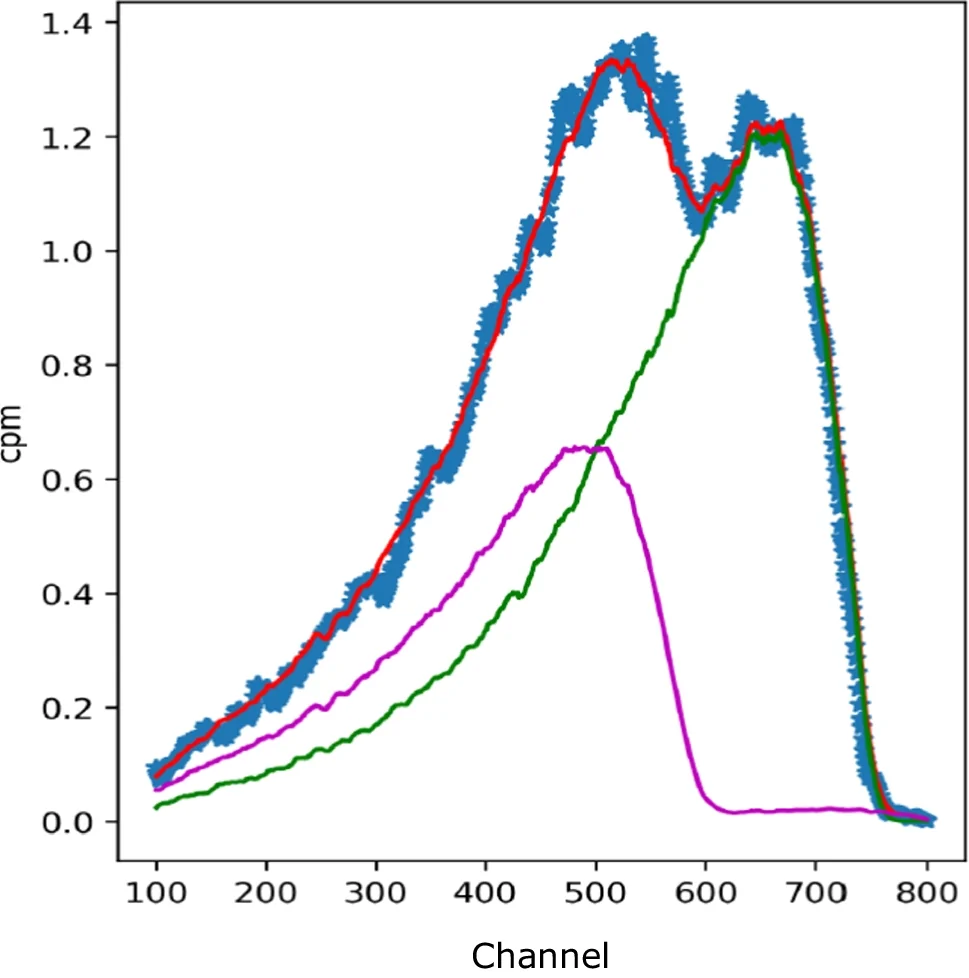
Experimental and reconstructed spectra for Sr89-90-3. The experimental (  ) and reconstructed (  ) spectra of the mixture, together with the individual spectra of $^{89}Sr$ (  ) and $^{90}Sr$ (  ) are shown

Regarding the $^{89}Sr/^{90}Sr$ mixtures, the errors calculated in the spectra reconstruction are shown in Fig. 5. It can be observed that using PLS, the error in the spectra reconstruction is generally smaller than that using LS. However, it has been observed that, when the proportion of $^{90}Sr$ is larger than that of $^{89}Sr$, PLS is not the most effective technique. In fact, this occurs for mixtures Sr89-90-4, Sr89-90-6, and Sr89-90-8, in which LS provides better results independently of the filtering technique. A more detailed study has to be undertaken for these mixtures. The maximum error observed in the spectra reconstruction corresponds to the mixture Sr89-90-3 using the Savitzky–Golay filter and LS, with a value of 4.57%. The minimum error is found in the mixture Sr89-90-8 with a value of 1.14%, using Laguerre or Legendre Polynomials and LS. The smallest average error is 1.84%, obtained using both Laguerre or Legendre Polynomials and PLS. All the error reconstruction values are presented in the supplementary material.

In fact, even for the maximum error in the reconstruction obtained, the agreement with the experimental spectrum is quite good. Figure 7 shows the output provided by the tool developed to reconstruct the spectra. In this figure, the measured and reconstructed spectra of the mixture together with the spectra of the two isotopes are shown. The figure corresponds to the mixture in which the maximum spectra reconstruction error (4.57%) was found for any of the test isotope mixtures analyzed, corresponding to mixture Sr89-90-3.

The tool also provides an estimation of the activity of each isotope present in the mixture. A relative bias between experimental and estimated activity has been calculated to assess the accuracy of the results. For $^{63}Ni/^{55}Fe$ mixtures, the maximum relative bias observed in the estimation of $^{63}Ni$ activity is -11.8% in mixture FENI-6, obtained using the Savitzky–Golay filter and LS, in which the proportion $^{63}Ni:^{55}Fe$ is 1:7 and the experimental activities of $^{63}Ni$ and $^{55}Fe$ are 4.939 Bq and 33.715 Bq, respectively. For the estimation of $^{55}Fe$ activity, the maximum relative bias observed is 41.4% for the mixture FENI-15 using the Savitzky–Golay filter and LS, being the experimental activities of $^{63}Ni$ and $^{55}Fe$ 40.294 Bq and 3.275 Bq, respectively. The high relative bias can be explained by the high proportion of nickel compared to iron. Note that the proportion $^{63}Ni:^{55}Fe$ is 12:1. In the field of radioactivity, relative biases of less than 20% are usually acceptable for estimating activity as proposed by the International Atomic Energy Agency in their proficiency tests [42]. The difference between experimental and estimated activities can be lowered using other filtering techniques, for example, Legendre polynomials, but a great decrease in the activity relative bias is observed when PLS is used instead of LS. Thus, using the Savitzky–Golay filter and PLS, the relative bias for $^{63}Ni$ in mixture FENI-6 is 0.641%, while for $^{55}Fe$ is -5.26% in sample FENI-15. In general, for all the $^{63}Ni/^{55}Fe$ mixtures analyzed, the use of PLS instead of LS improves both the error in the spectra reconstruction and the activity estimation for both isotopes.

The estimation of $^{14}C$ and $^{3}H$ activities also agrees well with the experimental data. The largest deviation for the estimation of $^{14}C$ activity is -2.66%, obtained in mixture H3C14-13 using the Savitzky–Golay filter and LS. In this mixture, the proportion $^{14}C:^{3}H$ is 1:8 and the activities of $^{14}C$ and $^{3}H$ are 5.630 Bq and 43.2 Bq, respectively. The activity of $^{14}C$ is much lower than the activity of $^{3}H$, which can result in larger relative bias when estimating the activity. One particularity observed in these mixtures is that the minimum difference in the estimation of $^{14}C$ activity is found for the mixture H3C14-3 also obtained using Savitzky–Golay and LS, with a difference of -0.005%. So in this case, the use of PLS does not improve the calculation of $^{14}C$ activity in the mixtures. A more in-depth study is needed. Regarding the estimation of $^{3}H$ activity, the largest difference found is 13.11% for the mixture H3C14-14 using the Fourier series and LS. The minimum value found is 0.064% for the mixture H3C14-13 using Legendre polynomials and PLS. In general, the estimation of $^{3}H$ activity is better when using PLS instead of LS.

For $^{89}Sr$, the largest relative bias in the activity estimation is -10.54% in mixture Sr89-90-6 using Savitzky–Golay and LS. In this mixture, the proportion $^{89}Sr:^{90}Sr$ is 1:10 and the activities are 4.229 Bq for $^{89}Sr$ and 42.321 Bq for $^{90}Sr$. The low activity of $^{89}Sr$ may be an explanation for the value of this result. The minimum relative bias found in $^{89}Sr$ activity is -0.13% for mixture Sr89-90-9 using Legendre polynomials and PLS. In this mixture, the proportion $^{89}Sr:^{90}Sr$ is 8:1 and the activities are 34.227 Bq for $^{89}Sr$ and 4.22 Bq for $^{90}Sr$. So, it is coherent that as the activity of $^{89}Sr$ is higher, the relative bias is lower. The use of PLS does not always improve the calculation of $^{89}Sr$ activity in the mixtures.

Finally, for $^{90}Sr$, the maximum relative bias obtained is 18.44% in mixture Sr89-90-9 using the Savitzky–Golay filter and LS. Note that the activity of this isotope in the mixture is quite low compared to the activity of $^{89}Sr$. Besides the activity, the shape of $^{89}Sr/^{90}Sr$ spectra (see Fig. 7) may contribute to a high relative bias compared to $^{63}Ni/^{55}Fe$ and $^{14}C/^{3}H$ spectra (see Fig. 6). When two or more isotopes overlap, the activity of the isotopes can be underestimated or overestimated more frequently if one or more of the peaks exhibits asymmetry towards the front (fronting peak) or an elongated tail towards the back (tailing peak). As can be seen in Fig. 7, the second peak ($^{89}Sr$) shows a fronting peak that can lead to an overestimation of the first peak ($^{90}Sr$). For this reason, the distortion of the shape makes it difficult to separate the signals correctly. On the other hand, the minimum activity difference for $^{90}Sr$ is 4.24%, obtained using Legendre polynomials and PLS in mixture Sr89-90-4. In this case, the proportion $^{89}Sr:^{90}Sr$ is 1:5 and the activities are 4.133 Bq for $^{89}Sr$ and 20.837 Bq for $^{90}Sr$. It is also noted that, in general, the use of PLS reduces the relative bias of the estimated activity.

## Conclusions

Nowadays, faster analysis processing times are necessary to effectively control the level of radioactivity in environmental samples. This is especially important for the decommissioning of nuclear power plants where the characterization of new isotopes not included in the existing Environmental Radiological Surveillance Programmes is required. In this framework, mixtures of $^{63}Ni/^{55}Fe$, $^{14}C/^{3}H$, and $^{89}Sr/^{90}Sr$, which are considered of interest in decommissioning or emergency situations, have been measured by liquid scintillation spectrometry and different deconvolution techniques have been developed to obtain the spectra of each isotope in the sample and to estimate their activity. In general, the results showed that the mathematical techniques implemented are capable of performing the spectra deconvolution and obtaining quite accurate estimations for the isotope activities in the mixtures, despite the large differences in the activity ratios. Regarding the reconstruction of the spectra, a maximum error of 4.57% is obtained, while the relative biases obtained in the estimation of the activities are less than 20% for all techniques applied to most of the test samples. For most of the mixtures analyzed, the combination of Legendre polynomials or Laguerre polynomials and partial least squares regression offers the best results for both the spectra reconstruction and the isotope activity estimation. However, further studies need to be conducted. For example, the number of isotopes in the mixture should be increased, as errors in reconstruction or activity estimation could increase. In the same way, a quantitative analysis of the shape of the spectrum considering skewness, peak overlap metrics, or channel-wise residuals should be performed. Another important parameter to be studied is quenching of the sample, which is similar for all the measurements done in this study. However, different SQP(E) values will probably affect the calculations. Future work will focus on analyzing how the proposed methodology behaves with real environmental samples, which are more difficult to manage than the samples studied in this work. In addition, the estimation of the activity uncertainty and limits of detections obtained with each method will be studied, considering the parameters affecting the filtering and spectra reconstruction techniques. The final product will be an open-source tool that will be available to environmental radioactivity laboratories through the Spanish Nuclear Safety Council (CSN, in Spanish). This tool will enable them to obtain their own deconvoluted liquid scintillation spectra after calibrating their specific equipment for the isotopes of interest.

## Supplementary Information

Below is the link to the electronic supplementary material.

Supplementary file 1 (pdf 98 KB)

## Data Availability

All data generated or analyzed during this study is included in the paper. The spectra of the calibration standards and mixtures are available at https://github.com/LRA-UPV/ACADE. For additional information, data will be available upon request.

## References

[1] ISO 10704: Water quality – gross alpha and gross beta activity – test method using thin source deposit. Standard, International Organization for Standardization, Geneva, Switzerland; 2019.

[2] ISO 11704: Water quality – gross alpha and gross beta activity – test method using liquid scintillation counting. Standard, International Organization for Standardization, Geneva, Switzerland; 2018.

[3] Stojković I, Todorović N, Nikolov J, Tenjović B. Establishment of rapid LSC method for direct alpha/beta measurements in waters. J Radioanal Nucl Chem. 2017;314:623–7.

[4] Sáez-Muñoz M, Ortiz J, Martorell S. Rapid evaluation of gross alpha and gross beta in water samples for emergency response. Radiat Phys Chem. 2020;171:108717.

[5] Sáez-Muñoz M, Bagán H, Tarancón A, García JF, Ortiz J, Martorell S. Rapid method for radiostrontium determination in milk in emergency situations using PS resin. J Radioanal Nucl Chem. 2018;315(3):543–55.

[6] dell’Oro D, Iammarino M, Bortone N, Mangiacotti M, Chiaravalle AE. Determination of radiostrontium in milk samples by ultralow- level liquid scintillation counting: a validated approach. Food Additives & Contaminants Part A. 2014;31(12):2014–21. 10.1080/19440049.2014.968883 25299737

[7] Iammarino M, dell’Oro D, Bortone N, Mangiacotti M, Chiaravalle AE. Optimisation and validation of a multi-matrix ultrasensible radiochemical method for the determination of radiostrontium in solid foodstuffs by liquid scintillation counting. Food Anal Methods. 2016;9:95–104.

[8] Marchesani G, Trotta G, De Felice P, Bortone N, Damiano R, Nicolini M, et al. Fast and sensitive radiochemical method for SR-90 determination in food and feed by chromatographic extraction and liquid scintillation counting. Food Anal Methods. 2022;15:1521–34.

[9] Sáez-Muñoz M, Bagán H, Tarancón A, García JF, Ortiz J, Carlos SSM. Rapid methods for radiostrontium determination in aerosol filters and vegetation in emergency situations using PS resin. J Radioan Nuclear Chemist. 2019;322:1397–408.

[10] Kim C, Al-Hamwi A, Törvényi A, Kis-Benedek G, Sansone U. Validation of rapid methods for the determination of radiostrontium in milk. Appl Radiat Isot. 2009;67(5):786–93. 19297175 10.1016/j.apradiso.2009.01.036

[11] Wold S, Sjöström M, Eriksson L. PLS-regression: a basic tool of chemometrics. Chemom Intell Lab Syst. 2001;58(2):109–30.

[12] Altzitzoglou T. Radioactivity determination of individual radionuclides in a mixture by liquid scintillation spectra deconvolution. Appl Radiat Isot. 2008;66(6–7):1055–61. 18359237 10.1016/j.apradiso.2008.02.052

[13] Fons-Castells J, Tent-Petrus J, Llauradó M. Simultaneous determination of specific alpha and beta emitters by LSC-PLS in water samples. J Environ Radioact. 2017;166:195–201. 27156160 10.1016/j.jenvrad.2016.04.035

[14] Grau Carles A, Grau Malonda A, et al. Spectral interpolation and unfolding to measure multi-labelled samples by liquid scintillation. Centro de Investigaciones Energeticas Medioambientales y Tecnologicas: Technical report; 1991.

[15] Malonda AG, Barquero LR, Carles AG. Radioactivity determination of 90Y, 90Sr and 89Sr mixtures by spectral deconvolution. Nucl Instrum Methods Phys Res, Sect A. 1994;339(1–2):31–7.

[16] Remetti R, Sessa A. Beta spectra deconvolution for liquid scintillation counting. J Radioanal Nucl Chem. 2011;287(1):107–11.

[17] Eisinberg A, Fedele G, et al. Discrete orthogonal polynomials on equidistant nodes. In: International Mathematical Forum, 2007;2:1007–1020.

[18] Savitzky A, Golay MJ. Smoothing and differentiation of data by simplified least squares procedures. Anal Chem. 1964;36(8):1627–39.

[19] Coma A, Tarancón A, Bagán H. Novel porous crosslinked plastic scintillation microspheres (pspscpsm) for radioactivity detection. Radiation Detection Technology and Methods, 2025.

[20] Neuer MJ. Spectral identification of a 90Sr source in the presence of masking nuclides using maximum-likelihood deconvolution. Nucl Instrum Methods Phys Res, Sect A. 2013;728:73–80.

[21] Bi H, Zhang Y, Wu B, Yuan D, Feng X, Shi Y. Study on reconstruction and analytical method of seawater radioactive gamma spectrum. Appl Radiat Isot. 2023;198:110853. 37216724 10.1016/j.apradiso.2023.110853

[22] Borovcová L, Hermannová M, Pauk V, Šimek M, Havlíček V, Lemr K. Simple area determination of strongly overlapping ion mobility peaks. Anal Chim Acta. 2017;981:71–9. 28693731 10.1016/j.aca.2017.05.003

[23] Renner G, Reuschenbach M. Critical review on data processing algorithms in non-target screening: challenges and opportunities to improve result comparability. Anal Bioanal Chem. 2023;415(18):4111–23. 37380744 10.1007/s00216-023-04776-7 PMC10328864

[24] Pilato V, Tola F, Martinez J, Huver M. Application of neural networks to quantitative spectrometry analysis. Nucl Instrum Methods Phys Res, Sect A. 1999;422(1–3):423–7.

[25] Chobola R, Chobola R, Mell P, Mell P, Daróczi L, Daróczi L, et al. Rapid determination of radiostrontium isotopes in samples of NPP origin. J Radioanal Nucl Chem. 2006;267(2):297–304.

[26] Li C, Liu S, Wang C, Jiang X, Sun X, Li M, et al. A new radionuclide identification method for low-count energy spectra with multiple radionuclides. Appl Radiat Isot. 2022;185:110219. 35413589 10.1016/j.apradiso.2022.110219

[27] Chen H, Zhang N, Du YH, Zhan XB, Li L, Cheng Z. Deep learning-based analysis and identification of single-particle mass spectra of bacteria. Anal Bioanal Chem. 2025;417:4265–77. 40542895 10.1007/s00216-025-05942-9

[28] Deng L, Xu G, Dai Y, Zhu H. A dual stream spectrum deconvolution neural network. IEEE Trans Industr Inf. 2021;18(5):3086–94.

[29] Li D-W, Hansen AL, Yuan C, Bruschweiler-Li L, Brüschweiler R. Deep picker is a deep neural network for accurate deconvolution of complex two-dimensional NMR spectra. Nat Commun. 2021;12(1):5229. 10.1038/s41467-021-25496-5 PMC8410766 34471142

[30] Molnar M, Reardon KP, Osborne C, Milić I. Spectral deconvolution with deep learning: removing the effects of spectral PSF broadening. Frontiers in Astronomy and Space Sciences. 2020;7:29.

[31] Schmid N, Bruderer S, Paruzzo F, Fischetti G, Toscano G, Graf D, et al. Deconvolution of 1D NMR spectra: a deep learning-based approach. J Magn Reson. 2023;347. 10.1016/j.jmr.2022.107357 36563418

[32] Xiaolin H, Ostergaard LF, Nielsen SP. Determination of and in nuclear waste samples using radiochemical separation and liquid scintillation counting. Analytica Chimica Acta 2005;535.

[33] Verdú G, Ginestar D, Vidal V. Dynamics reconstruction using orthonormal functions. Appl Math Lett. 1999;12(8):39–43.

[34] Lee YW. Statistical Theory of Communication, vol. 284. New York: Wiley; 1960.

[35] Steinier J, Termonia Y, Deltour J. Smoothing and differentiation of data by simplified least square procedure. Anal Chem. 1972;44(11):1906–9. 22324618 10.1021/ac60319a045

[36] Nocedal J, Wright SJ. Numerical Optimization. Springer, 2006.

[37] Haaland DM, Thomas EV. Partial least-squares methods for spectral analyses. 1. relation to other quantitative calibration methods and the extraction of qualitative information. Analytical chemistry 1988;60(11):1193–1202.

[38] Geladi P, Kowalski BR. Partial least-squares regression: a tutorial. Anal Chim Acta. 1986;185:1–17.

[39] Fons-Castells J, Llopart-Babot I, Franco-Curtido JA, Tent-Petrus J, Rigol A, Llaurado M. Declab: a software for liquid scintillation spectra deconvolution. J Radioanal Nucl Chem. 2022;331(8):3275–82.

[40] Wold H. Estimation of principal components and related models by iterative least squares. Multivariate analysis, 1966;391–420.

[41] Wold H. Nonlinear iterative partial least squares (nipals) modelling: some current developments. In: Multivariate analysis-III, 1973;383–407. Elsevier.

[42] Agency IAE. Summary report of iaea-terc-2024-01 world wide open proficiency test exercise (terrestrial environmental radiochemistry laboratory, international atomic energy agency). Technical report, International Atomic Energy Agency (October 17, 2024).

