# Supplementary material for: Advancing liquid scintillation spectra deconvolution for radiological characterization: comparative techniques for multi-isotope activity assessment
Source: Anal Bioanal Chem. 2026 Feb 20;418(18):5907–19. doi: 10.1007/s00216-026-06392-7 (PMC13541852; doi:10.1007/s00216-026-06392-7)
Supplement: Supplementary file 1 [file 216_2026_6392_MOESM1_ESM.pdf]

Supplementary information:. The following Tables are presented here as supplementary information of the manuscript *Advancing liquid scintillation spectra deconvolution for radiological characterization: comparative techniques for multi-isotope activity assessment.*

Table 1 $^{63}Ni/^{55}Fe$ mixtures.

Mixture ID	$^{63}Ni:^{55}Fe$ Ratio	^{63}Ni Activity (Bq)	^{55}Fe Activity (Bq)
FENI-1	1.5:1	4.859	3.272
FENI-2	1:1.5	4.931	6.977
FENI-3	3:1	9.832	3.272
FENI-4	1:3.5	4.927	16.935
FENI-5	7:1	24.822	3.280
FENI-6	1:7	4.934	33.715
FENI-7	15:1	48.549	3.288
FENI-8	1:2	4.836	10.316
FENI-9	5:1	14.886	3.247
FENI-10	1:3	4.885	13.628
FENI-11	6:1	19.709	3.279
FENI-12	1:4	4.882	20.106
FENI-13	9:1	29.476	3.284
FENI-14	1:6	4.888	27.126
FENI-15	12:1	40.294	3.275

Table 2 $^{14}C/^{3}H$ mixtures.

Mixture ID	$^{14}C/^{3}H$ Ratio	^{14}C Activity (Bq)	^{3}H Activity (Bq)
H3C14-1	1:1	5.666	5.010
H3C14-2	2:1	9.303	5.011
H3C14-3	1:2	5.648	10.229
H3C14-4	3:1	14.895	5.014
H3C14-5	1:2.5	5.722	14.352
H3C14-6	4:1	20.322	5.012
H3C14-7	1:4	5.703	21.385
H3C14-8	5:1	25.951	5.013
H3C14-9	1:4.5	5.648	24.829
H3C14-10	6:1	29.643	5.011
H3C14-11	1:5	5.593	28.632
H3C14-12	8:1	41.566	5.013
H3C14-13	1:8	5.630	43.200
H3C14-14	10:1	50.555	5.014
H3C14-15	1:9	5.648	49.945

Table 3 $^{89}Sr/^{90}Sr$ mixtures.

Mixture ID	$^{89}Sr/^{90}Sr$ Ratio	^{89}Sr Activity (Bq)	^{90}Sr Activity (Bq)
Sr89-90-1	1:1	4.330	4.294
Sr89-90-2	1:2	3.459	6.897
Sr89-90-3	2:1	5.233	2.594
Sr89-90-4	1:5	4.133	20.837
Sr89-90-5	5:1	21.779	4.386
Sr89-90-6	1:10	4.229	42.321
Sr89-90-7	10:1	40.730	4.046
Sr89-90-8	1:8	4.358	34.835
Sr89-90-9	8:1	34.227	4.229

Table 4 $^{63}Ni/^{55}Fe$ mixtures in percentage.

	LS				PLS			
	Fourier	Laguerre	Legendre	Savitzky–Golay	Fourier	Laguerre	Legendre	Savitzky-Golay
FENI-1	1.35	1.17	1.17	2.63	0.94	0.63	0.96	2.47
FENI-2	2.03	1.87	1.88	2.85	0.65	0.50	0.56	1.99
FENI-3	1.65	1.47	1.50	2.30	1.15	0.82	0.76	1.83
FENI-4	2.44	2.42	2.37	3.45	0.59	0.47	0.64	2.53
FENI-5	2.19	2.13	2.01	2.49	1.62	1.52	1.41	1.81
FENI-6	2.81	2.79	2.77	3.07	0.75	0.67	0.78	1.73
FENI-7	0.97	0.88	0.87	1.46	0.48	0.33	0.37	1.15
FENI-8	1.83	1.63	1.62	2.35	0.70	0.45	0.41	1.91
FENI-9	1.18	1.08	1.05	1.68	0.81	0.48	0.36	1.39
FENI-10	2.08	2.05	2.02	2.51	0.55	0.52	0.60	1.79
FENI-11	2.14	2.09	2.00	2.54	1.58	1.49	1.39	1.88
FENI-12	1.99	1.99	1.98	2.51	0.90	0.60	0.58	2.30
FENI-13	1.74	1.66	1.59	2.10	1.15	1.06	1.02	1.40
FENI-14	2.71	2.65	2.61	3.29	0.75	0.42	0.44	2.10
FENI-15	0.96	0.88	0.78	1.43	0.78	0.64	0.58	1.35
Average	1.87	1.78	1.75	2.44	0.89	0.71	0.72	1.84

Table 5 $^{14}C/^{3}H$ mixtures in percentage.

	LS				PLS			
	Fourier	Laguerre	Legendre	Savitzky–Golay	Fourier	Laguerre	Legendre	Savitzky-Golay
H3C14-1	2.23	1.94	1.92	2.99	1.59	1.11	1.13	2.50
H3C14-2	1.62	1.56	1.61	2.23	1.17	1.08	1.10	1.91
H3C14-3	2.32	2.25	2.27	2.74	1.30	1.16	1.04	1.89
H3C14-4	1.03	0.92	0.98	1.82	0.83	0.70	0.66	1.73
H3C14-5	1.84	1.70	1.76	2.60	0.94	0.64	0.60	2.04
H3C14-6	1.07	0.88	0.92	1.76	0.69	0.38	0.40	1.56
H3C14-7	2.99	2.96	2.96	3.28	1.34	1.23	1.26	1.81
H3C14-8	0.99	0.92	0.95	1.52	0.60	0.49	0.51	1.29
H3C14-9	1.52	1.52	1.51	1.90	0.96	0.94	0.92	1.39
H3C14-10	1.88	1.81	1.83	2.11	1.21	1.12	1.15	1.55
H3C14-11	1.58	1.46	1.48	2.34	0.88	0.66	0.71	1.93
H3C14-12	1.78	1.63	1.68	2.02	1.14	0.91	0.92	1.50
H3C14-13	3.11	2.97	2.95	3.32	1.40	0.99	0.97	1.79
H3C14-14	2.21	2.17	2.18	2.47	1.55	1.50	1.47	1.89
H3C14-15	2.17	2.13	2.11	2.41	0.91	0.82	0.80	1.34
Average	1.89	1.79	1.81	2.37	1.10	0.92	0.91	1.74

Table 6 $^{89}Sr/^{90}Sr$ mixtures in percentage.

	LS				PLS			
	Fourier	Laguerre	Legendre	Savitzky–Golay	Fourier	Laguerre	Legendre	Savitzky–Golay
Sr89-90-1	2.81	2.81	2.82	4.02	2.56	2.48	2.50	3.78
Sr89-90-2	1.75	1.62	1.62	3.08	1.98	1.55	1.55	3.06
Sr89-90-3	2.99	2.74	2.73	4.57	2.60	2.31	2.31	4.32
Sr89-90-4	1.40	1.29	1.29	2.29	1.60	1.57	1.57	2.44
Sr89-90-5	2.79	2.74	2.74	3.27	2.15	2.08	2.08	2.70
Sr89-90-6	1.46	1.38	1.39	1.83	1.96	1.90	1.90	2.22
Sr89-90-7	2.49	2.51	2.50	2.94	1.72	1.74	1.74	2.29
Sr89-90-8	1.31	1.14	1.14	1.93	1.75	1.63	1.62	2.25
Sr89-90-9	2.10	1.97	1.95	2.71	1.48	1.30	1.27	2.24
Average	2.12	2.02	2.02	2.96	1.98	1.84	1.84	2.81

Table 7 ^{63}Ni relative activity bias in percentage.

	LS				PLS			
	Fourier	Laguerre	Legendre	Savitzky–Golay	Fourier	Laguerre	Legendre	Savitzky-Golay
FENI-1	-0.0753	-0.07742	-0.0692	0.134	0.935	0.949	0.945	1.45
FENI-2	-2.23	-2.28	-2.25	-2.71	-0.751	-0.763	-0.769	-0.0046
FENI-3	-0.818	-0.815	-0.818	-0.332	0.0126	0.0218	0.0218	0.421
FENI-4	-3.32	-3.34	-3.32	-5.52	-0.602	-0.594	-0.594	0.782
FENI-5	-1.64	-1.64	-1.64	-0.989	-0.935	-0.925	-0.923	-0.585
FENI-6	-6.56	-6.62	-6.59	-11.8	-1.81	-1.80	-1.81	0.641
FENI-7	1.32	1.32	1.32	1.32	2.01	2.00	2.00	2.33
FENI-8	-0.833	-0.862	-0.800	-0.800	1.12	1.12	1.11	2.12
FENI-9	-1.42	-1.41	-1.41	-1.43	-0.665	-0.652	-0.647	-0.293
FENI-10	-2.95	-3.00	-2.96	-2.97	-0.649	-0.6.39	-0.6.43	0.528
FENI-11	-0.784	-0.788	-0.774	-0.792	-0.0455	-0.0455	-0.0379	0.308
FENI-12	-2.4	-2.38	-2.37	-2.39	0.781	0.818	0.826	2.42
FENI-13	-1.18	-1.19	-1.19	-1.19	-0.478	-0.478	-0.477	-0.140
FENI-14	-4.97	-5.00	-4.99	-4.92	-0.906	-0.908	-0.904	1.18
FENI-15	1.43	1.43	1.43	1.42	2.12	2.13	2.13	2.46

Table 8 ^{55}Fe relative activity bias in percentage.

	LS				PLS			
	Fourier	Laguerre	Legendre	Savitzky–Golay	Fourier	Laguerre	Legendre	Savitzky-Golay
FENI-1	1.25	1.2	1.07	1.25	-2.88	-3.01	-3.01	-4.99
FENI-2	3.36	3.46	3.43	3.36	1.13	1.15	1.15	-0.0274
FENI-3	5.04	5.02	5.02	5.07	-2.76	-2.91	-2.91	-6.70
FENI-4	0.297	0.318	0.290	0.274	-0.941	-0.957	-0.957	-1.62
FENI-5	28.0	27.8	27.7	28.0	9.33	8.95	8.95	-0.147
FENI-6	-0.118	-0.0988	-0.116	-0.128	-1.01	-1.02	-1.02	-1.51
FENI-7	33.5	33.8	33.9	33.5	-3.56	-3.53	-3.53	-22.0
FENI-8	1.43	1.47	1.48	1.40	-0.286	-0.291	-0.291	-1.20
FENI-9	13.5	13.3	13.2	13.4	1.93	1.63	1.63	2.00
FENI-10	-0.0996	-0.0812	-0.0937	-0.0996	-1.51	-1.53	-1.53	-2.26
FENI-11	18.4	18.5	18.3	18.4	3.38	3.35	3.35	-4.10
FENI-12	0.803	0.774	0.757	0.773	-0.371	-0.418	-0.418	-1.03
FENI-13	36.1	36.3	36.1	36.2	13.9	13.8	13.8	2.92
FENI-14	1.67	1.70	1.68	1.64	0.682	0.681	0.681	0.124
FENI-15	41.4	41.2	41.2	41.4	10.4	10.0	10.0	-5.26

Table 9 ^{14}C relative activity bias in percentage.

	LS				PLS			
	Fourier	Laguerre	Legendre	Savitzky–Golay	Fourier	Laguerre	Legendre	Savitzky-Golay
H3C14-1	0.469	0.469	0.469	0.443	0.785	0.788	0.786	0.781
H3C14-2	-0.741	-0.748	-0.744	-0.745	-0.490	-0.488	-0.489	-0.495
H3C14-3	-1.719	-1.678	-1.678	-1.673	-1.166	-1.168	-1.168	-1.170
H3C14-4	0.013	0.013	0.013	-0.005	0.212	0.216	0.215	0.210
H3C14-5	-0.510	-0.488	-0.488	-0.507	0.164	0.169	0.169	0.154
H3C14-6	0.122	0.121	0.113	0.120	0.311	0.314	0.314	0.307
H3C14-7	-0.308	-0.308	-0.308	-0.308	0.606	0.606	0.607	0.604
H3C14-8	-0.081	-0.084	-0.084	-0.091	0.090	0.093	0.092	0.087
H3C14-9	-0.571	-0.605	-0.605	-0.612	0.498	0.498	0.498	0.494
H3C14-10	0.381	0.381	0.381	0.376	0.557	0.558	0.558	0.554
H3C14-11	0.392	0.420	0.428	0.387	1.647	1.659	1.657	1.656
H3C14-12	0.199	0.202	0.205	0.195	0.367	0.372	0.371	0.364
H3C14-13	-2.656	-2.637	-2.637	-2.658	-0.917	-0.901	-0.903	-0.913
H3C14-14	-0.249	-0.247	-0.247	-0.254	-0.081	-0.079	-0.079	-0.086
H3C14-15	0.212	0.243	0.243	0.221	2.277	2.288	2.288	2.288

Table 10 ^{3}H relative activity bias in percentage.

	LS				PLS			
	Fourier	Laguerre	Legendre	Savitzky–Golay	Fourier	Laguerre	Legendre	Savitzky-Golay
H3C14-1	1.656	1.667	1.667	1.742	0.385	0.385	0.385	0.409
H3C14-2	4.062	4.136	4.090	4.187	2.055	2.055	2.079	2.111
H3C14-3	1.410	1.426	1.426	1.444	0.775	0.775	0.789	0.807
H3C14-4	3.207	3.218	3.218	3.220	-0.066	-0.066	-0.084	-0.076
H3C14-5	3.138	3.126	3.126	3.136	2.624	2.624	2.621	2.636
H3C14-6	4.971	4.923	4.921	4.948	0.482	0.482	0.458	0.468
H3C14-7	1.385	1.386	1.386	1.390	1.032	1.032	1.033	1.035
H3C14-8	2.055	2.029	2.029	2.085	-3.564	-3.564	-3.596	-3.570
H3C14-9	1.912	1.923	1.923	1.937	1.564	1.564	1.565	1.568
H3C14-10	7.556	7.566	7.566	7.631	1.133	1.133	1.111	1.157
H3C14-11	0.729	0.707	0.709	0.710	0.402	0.402	0.389	0.380
H3C14-12	9.311	9.205	9.170	9.213	0.310	0.310	0.210	0.230
H3C14-13	0.276	0.278	0.278	0.280	0.068	0.068	0.064	0.066
H3C14-14	13.105	13.070	13.070	13.072	2.038	2.038	1.998	2.082
H3C14-15	1.133	1.135	1.135	1.132	0.911	0.911	0.906	0.903

Table 11 ^{89}Sr relative activity bias in percentage.

	LS				PLS			
	Fourier	Laguerre	Legendre	Savitzky–Golay	Fourier	Laguerre	Legendre	Savitzky–Golay
Sr89-90-1	-1.423	-1.415	-1.429	-1.403	-0.692	-0.659	-0.650	-0.680
Sr89-90-2	0.572	0.442	0.579	0.457	1.712	1.714	1.720	1.723
Sr89-90-3	3.825	3.865	3.844	3.809	4.392	4.398	4.394	4.379
Sr89-90-4	-6.915	-6.972	-6.899	-6.862	-4.340	-4.359	-4.366	-4.275
Sr89-90-5	1.482	1.482	1.484	1.485	1.856	1.855	1.855	1.852
Sr89-90-6	-10.543	-10.456	-10.368	-10.540	-5.551	-5.503	-5.468	-5.577
Sr89-90-7	1.393	1.386	1.385	1.381	1.701	1.696	1.697	1.693
Sr89-90-8	-8.780	-8.879	-8.856	-8.779	-4.848	-4.901	-4.940	-4.779
Sr89-90-9	-0.436	-0.434	-0.449	-0.462	-0.119	-0.120	-0.133	-0.142

Table 12 ^{90}Sr relative activity bias in percentage.

	LS				PLS			
	Fourier	Laguerre	Legendre	Savitzky–Golay	Fourier	Laguerre	Legendre	Savitzky–Golay
Sr89-90-1	14.439	14.436	14.438	14.447	13.777	13.735	13.735	13.777
Sr89-90-2	10.749	10.796	10.764	10.803	10.327	10.314	10.314	10.317
Sr89-90-3	19.487	19.467	19.498	19.536	18.199	18.211	18.241	18.295
Sr89-90-4	5.123	5.148	5.135	5.117	4.808	4.812	4.814	4.787
Sr89-90-5	17.804	17.817	17.806	17.849	15.380	15.392	15.392	15.401
Sr89-90-6	4.544	4.541	4.533	4.552	4.249	4.246	4.243	4.253
Sr89-90-7	18.309	18.379	18.350	18.339	13.725	13.841	13.833	13.811
Sr89-90-8	3.889	3.896	3.899	3.874	3.593	3.596	3.600	3.574
Sr89-90-9	18.267	18.340	18.377	18.438	14.559	14.611	14.715	14.741
